# Ethnic disparities in children’s oral health: findings from a population-based survey of grade 1 and 2 schoolchildren in Alberta, Canada

**DOI:** 10.1186/s12903-017-0444-8

**Published:** 2018-01-04

**Authors:** Congshi Shi, Peter Faris, Deborah A. McNeil, Steven Patterson, Melissa L. Potestio, Salima Thawer, Lindsay McLaren

**Affiliations:** 1 Department of Community Health Sciences, University of Calgary, TRW3, 3280 Hospital Dr. NW, Calgary, AB T2N 4Z6 Canada; 2 School of Dentistry, University of Alberta, Edmonton, AB Canada; 3 Alberta Health Services, Calgary, AB Canada; 4 Faculty of Nursing, University of Calgary, Calgary, AB Canada

**Keywords:** Oral health, Dental caries, Health status disparities, Ethnicity, Minority groups, Children

## Abstract

**Background:**

Although oral health has improved remarkably in recent decades, not all populations have benefited equally. Ethnic identity, and in particular visible minority status, has been identified as an important risk factor for poor oral health. Canadian research on ethnic disparities in oral health is extremely limited. The aim of this study was to examine ethnic disparities in oral health outcomes and to assess the extent to which ethnic disparities could be accounted for by demographic, socioeconomic and caries-related behavioral factors, among a population-based sample of grade 1 and 2 schoolchildren (age range: 5-8 years) in Alberta, Canada.

**Methods:**

A dental survey (administered during 2013-14) included a mouth examination and parent questionnaire. Oral health outcomes included: 1) percentage of children with dental caries; 2) number of decayed, extracted/missing (due to caries) and filled teeth; 3) percentage of children with two or more teeth with untreated caries; and 4) percentage of children with parental-ratings of fair or poor oral health. We used multivariable regression analysis to examine ethnic disparities in oral health, adjusting for demographic, socioeconomic and caries-related behavioral variables.

**Results:**

We observed significant ethnic disparities in children’s oral health. Most visible minority groups, particularly Filipino and Arab, as well as Indigenous children, were more likely to have worse oral health than White populations. In particular, Filipino children had an almost 5-fold higher odds of having severe untreated dental problems (2 or more teeth with untreated caries) than White children. Adjustment for demographic, socioeconomic, and caries-related behavior variables attenuated but did not eliminate ethnic disparities in oral health, with the exception of Latin American children whose outcomes did not differ significantly from White populations after adjustment.

**Conclusions:**

Significant ethnic disparities in oral health exist in Alberta, Canada, even when adjusting for demographic, socioeconomic and caries-related behavioral factors, with Filipino, Arab, and Indigenous children being the most affected.

**Electronic supplementary material:**

The online version of this article (10.1186/s12903-017-0444-8) contains supplementary material, which is available to authorized users.

## Background

Although dental caries, also known as cavities or tooth decay, is largely preventable, it remains the preeminent oral disease of childhood and adolescence [1]. Dental caries negatively affects children’s oral health-related quality of life, causing disturbances such as pain, impaired speaking, eating and sleeping disruptions [2, 3] and poor school performance [4].

In recent decades, remarkable progress has been made in improving oral health; however, not all populations have benefited equally. Dental caries is disproportionately experienced by socially disadvantaged individuals, such as those of lower socioeconomic position, and/or within certain ethnic groups [1, 5, 6]. Canadians from lower income families have almost two times worse dental health outcomes compared to higher income Canadians [7]. One important contributor to poor dental health among socially disadvantaged populations in Canada is limited access to dental care. Most dental services in Canada are privately financed. Although a small proportion of dental services are federally or provincially funded, not all vulnerable Canadians are eligible [8].

The present study focuses on ethnicity. An ethnic group may be defined as a category of people who identify with each other based on similarities, such as common language, ancestral, social, cultural, or national experiences [9]. According to Statistics Canada, the White population refers to those who identify as Caucasian in race or white in color, whereas the visible minority population includes persons who are non-Caucasian in race or non-white in colour and who do not report being Indigenous [10]. Ethnicity is a significant determinant of oral health in numerous countries, and visible minority status is a well-documented risk factor, or marker of risk, for poor oral health [11].

There is important theoretical and empirical literature on ethnic health inequalities, which considers how social exclusionary processes such as labour market segregation, unemployment and income inequality, and poverty disproportionately impact racialized and immigrant groups, and translate into health disparities [12]. Published studies from the United States (U.S.) have highlighted several factors as relevant for explaining ethnic disparities in oral health, including socioeconomic conditions, health literacy, educational attainment, dental insurance, language barriers, and cultural characteristics [13–15]. Some behaviors were also identified as relevant to understanding or explaining ethnic inequalities in oral health, such as poor dental hygiene behavior, sugary dietary habits and early childhood bottle-feeding practices [16–18].

Several studies have examined the association between ethnicity and oral health outcomes, though they differ in how they define ethnicity. Research using population-based samples from the United States (U.S.), United Kingdom (U.K.) and elsewhere has documented that, in general, visible minority populations experience poorer oral health compared to White populations. In the U.S., research has focused on health disparities between White, Black, and non-white Hispanic children under 18 years old and found that Black and non-white Hispanic children experienced poorer dental health than White children. Further, these inequalities were found to be largely attributable to socioeconomic determinants such as income and health insurance [19–21]. In the U.K., research has shown that among preschool children, certain ethnic minority groups, such as Asian Pakistani, Asian Bangladeshi, Black and Caribbean populations, experienced significantly higher rates of caries than their White British counterparts [22]. On the other hand, in a recent study using a national U.K. sample, better oral health was observed among non-White compared to White adult groups, and these differences were partially explained by reported differences in dietary sugar [23]. While patterns of ethnic inequalities in oral health observed in other countries (e.g., the U.S., the U.K.) may apply to the Canadian situation, they also may not because of important differences in the ethnic composition of the countries. In the U.S., much quantitative health research classifies people by race, using large categories such as White, Black, and Hispanic [24]. In Canada, in contrast, a more common classification is White, Indigenous, and visible minorities, with examples of the latter category including Filipino, Chinese, and South Asian (e.g., Indian, Bangladeshi) [10].

There is very little Canadian research on ethnic disparities in oral health, which in part reflects data limitations – namely, the limited number of population-based surveys that contain data on ethnic identity and clinician-assessed oral health outcomes. The limited Canadian research on this subject constitutes an important knowledge gap. Canada is one of the most ethnically diverse countries in the world. The proportion of visible minorities has increased significantly over the past few decades, both in Canada and within the province of Alberta, where our study is situated. By 2031, Statistics Canada projects that close to 30% of the Canadian population and 25% of Alberta’s population will be a member of a visible minority group [25]. Therefore, greater understanding of ethnic disparities in oral health in Alberta and Canada is important to inform future policies and programs.

The primary objective of this study was to examine, amongst a population-based sample of grade 1 and 2 schoolchildren, ethnic disparities in oral health. The secondary objective was to assess the extent to which ethnic disparities in oral health could be accounted for by demographic, socioeconomic, and caries-related behavioral factors.

## Methods

### Data source

Data analyzed for this paper were drawn from a larger cross-sectional study, the objective of which was to explore the implications of cessation of community water fluoridation for children’s dental caries experience [26, 27]. The target population was children in grades 1 and 2 attending school in the Public or Catholic school system in the cities of Calgary and Edmonton, Alberta, Canada. Calgary and Edmonton are the two largest cities in the province of Alberta and are both large urban centres with diverse demographic profiles.

Data were collected during 2013/14. A multistage probability sampling strategy was used. Within each city, schools were stratified into quartiles based on the median household income of the neighbourhood in which the school was located, and schools were selected randomly from each quartile (strata). Within sampled schools, all children of eligible grades were invited to participate. In the end, 125 (out of 220) and 117 (out of 218) schools participated in Calgary and Edmonton respectively. The overall school-level response rates were 57.3% and 54.1% in Calgary and Edmonton, respectively. The overall student-level response rates within participating schools were 49.1% and 47.0% in Calgary and Edmonton, respectively.

Data collection included both an mouth examination and a structured Alberta Oral Health Questionnaire completed by respondents’ parents. The total number of records in the original dataset was 8641, of which 7548 students had mouth examination data and 8419 students’ parents completed the parent questionnaire. A total of 6884 students had data from both the parent questionnaire and the mouth examination (with both primary and permanent teeth), and constituted the initial sample for the present study.

The mouth examination was conducted at school by 5 trained and calibrated assessment teams (3 teams in Calgary and 2 teams in Edmonton), each consisting of a registered dental hygienist and a clerk. The mouth examination followed the caries diagnostic criteria [28, 29] to yields caries experience using the traditional deft/DMFT index (i.e., decayed, extracted/missing (due to decay), and filled teeth (primary and permanent)). All five assessment teams were trained together in the protocol and received both theoretical and hands-on calibration. Specifically, the theoretical calibration occurred for everyone together; the hands-on calibration occurred in two locations (all Calgary teams and all Edmonton teams), 1 day apart, by the same calibrator. The calibrator is a public health dentist with considerable experience in survey calibration and extensive knowledge and background in survey methodology. Signed parental consent and verbal child assent were secured.

Data collection also included a hard-copy questionnaire, which was distributed at school, completed by parents, and returned via mail. The questionnaire included items in the following domains: parent reports of children’s dental health; visits to dental health professionals; child’s food and beverage consumption; child’s use of fluoride; place of residence and drinking water source; and socio-demographic information. Specific variables from the questionnaire that were used in the present study are described below.

### Variables

#### Oral health outcomes

The deft/DMFT index is a commonly used index for cumulative measures of dental caries in populations and represents the total number of decayed (cavitated) (d/D), extracted/missing (e/M, due to decay) and filled (f/F) teeth. The lowercase letters (deft) refer to primary teeth, whereas the capitalized letters (DMFT) refer to permanent teeth.

We used four oral health outcomes. Three of these were “clinician-assessed” based on the results from the mouth examination: 1) caries prevalence, or the percentage of children with some caries experiences (i.e., percent with deft/DMFT >0); 2) number of decayed, extracted/missing (due to decay) and filled teeth (deft/DMFT); and 3) the percentage of children with two or more teeth (primary or permanent) with untreated caries (i.e., percent with 2 or more d or D). The fourth outcome was “parent perception” based on questionnaire responses (“In general, how would you rate the health of your child’s mouth, including his/her teeth, tongue, gums, lips, and jaw joint?” [excellent / very good / good / fair / poor]). For this outcome, we used the percentage of children rated by their parents as having fair or poor oral health.

#### Primary independent variable: Ethnicity

Ethnic identity, as reported on the parent questionnaire, was the primary independent variable in this study. Additional file 1: Appendix A presents the question used, which was drawn from Cycle 1 of the Canadian Health Measures Survey (CHMS), household interview [30]. Statistics Canada questionnaire items, including those used in the Canadian Health Measures Survey, underwent extensive field testing prior to use, thus enhancing their reliability and validity [31]. Briefly, parents were asked to select all that apply amongst 15 “ethno-cultural background” groups (see Additional file 1: Appendix A). For those whose parents selected “other-specify” and provided a written response, we re-classified ethnic group into an existing category when possible. For example, we reclassified one written response of “Barbados” to the existing category of Latin America, based on geographic location. Other responses of “other” were either excluded if too few in number, or classified as “mixed ethnic group” (see below).

We restricted our analysis to ethnic groups with a sample size of at least 100; these included: White, South Asian, Filipino, Chinese, Black, Arab, Latin American, and Indigenous. The minimum of 100, though somewhat arbitrary, gave us balance between the number of ethnic groups available to examine (a higher minimum number would have meant fewer groups) and reasonable sample size for multivariate analysis. We combined First Nations, Métis and Inuit into one group of Indigenous.^1^ Though the sample size was slightly smaller than 100 (*n* = 95), we included Indigenous children in our study because of the unique importance of that identity in Canada [32, 33]. It is important to note that the Indigenous children included in this study live in two urban areas (Calgary and Edmonton); Indigenous children living on reserves were not part of the sampling frame. Finally, we aggregated all children whose parents selected more than one (two or more) ethnic group into one group, which we called “mixed ethnic group”.

#### Caries-related behavioral variables

We considered three caries-related behavioral variables, all of which were included in the parent questionnaire. All caries-related behavioral variables were dichotomous for the purpose of analysis, which reflects our desire for a balance between capturing some differential exposure and retaining a parsimonious model (avoiding multiple categories of multiple covariates).

The first variable is tooth brushing: less than twice per day versus twice or more per day, based on recommendations by the Canadian Dental Association and dental public health organizations [7]. Second, we considered children’s parent-reported consumption frequency of sugar-sweetened beverages (regular [non-diet] soft drinks, soda pop, sports drinks, fruit-flavoured drinks), which has been shown to increase the risk of developing dental caries [1]. We constructed a two-level indicator: low/medium (less than 4 times per week) versus high (4 or more times per week). Third, we considered regular visits to a dental professional, and constructed a two-level indicator: one or more routine dental visits (including preventive or treatment-based visits, excluding emergency visits) in the past year, versus no routine dental visit in the past year.

#### Socioeconomic variables

We examined three indicators of socioeconomic circumstances. As with the caries-related behavioral variables, all socioeconomic variables were dichotomous: 1) highest level of household educational attainment (higher than high school diploma versus high school diploma or less); 2) dwelling ownership (owned with or without mortgage versus non-owned); and 3) dental insurance status (uninsured versus insured with public or private insurance). Although dental insurance is not a direct marker of socio-economic status, we opted to include it because it gives an indication of resources that permit some access to dental services.

#### Other covariates

Other variables included in analyses were children’s age in years, sex (male, female), and city (Calgary or Edmonton).

### Data analysis

All data analyses were carried out using STATA 14 survey commands. Two-tailed *p* values of less than 0.05 were considered statistically significant. Sampling weights were developed to account for the clustered sampling design (probability weights) and to account for imbalances in participants’ socioeconomic status (post stratification weights, with post-strata corresponding to the median household income of the neighborhood [census dissemination area] in which the school was located).

First, to characterize the sample, the distribution of oral health outcomes, socioeconomic and caries-related behaviors variables was examined across all ethnic groups. For all comparisons, we used the chi-square test (for categorical variables) with Rao-Scott corrections [34] to account for the complex survey design, and the Kruskal Wallis test (for skewed count variable) to determine whether the differences between ethnic groups were statistically significant.

Then, multivariable regression analysis was used to achieve the main study objectives (i.e., to examine the effect of ethnicity on oral health, and to assess whether or the extent to which the effect could be accounted for by demographic, socioeconomic, and some caries-related behavioral variables). We used binary logistic regression for the following outcomes: children with dental caries (outcome 1), children with ≥2 teeth with untreated caries (outcome 3), and children with parent-rated fair or poor oral health (outcome 4). Because caries experience (count of deft/DMFT, outcome 2) is an over-dispersed count variable with excess zeroes, the zero-inflated negative binomial (ZINB) regression was used, in which the same sets of independent variables were used in both the counting and inflated model components in ZINB regression [35]. The odds ratios (ORs) from the logistic regression component of the ZINB regression were identical to the inverse of the ORs from the binary logistic regression for caries prevalence (outcome 1); therefore, we do not present the binary logistic regression output in ZINB.

For each of the four oral health outcomes, we ran three regression models. Each successive model contained an additional block of covariates, which permitted us to see whether/to what extent the effect of ethnicity on oral health changed as additional blocks of covariates were added (i.e., demographic [model 1], socioeconomic [model 2], and caries-related behaviors [model 3]). Each successive model included the covariate block(s) from the previous models, such that model 3 is the fully-adjusted model. We also report fit indices (c statistic, and Root Mean Square Error) for our fully adjusted models.

### Ethics

The original study received approval from the Conjoint Health Research Ethics Board at the University of Calgary (ID E-25219) and the Health Research Ethics Board at the University of Alberta (ID Pro00037808).

## Results

### Description of the sample: Missing data, ethnic identity distribution, and comparison with national population estimates

Of the 6884 participants who had the data from both parent questionnaire and mouth examination (with both primary and permanent teeth), our analytic sample size was *n* = 5600 (see Additional file 2: Figure S1 for a flow chart of exclusions). There are some similarities and some differences between those with complete data (*n* = 5600) and those with missing covariate data (*n* = 792, which is the most important reason for excluding participants, see Additional file 2: Figure S1). Specifically, compared to those with missing covariate data, those with complete data had a higher percentage of White (51.8% versus 31.8%), and lower percentages of South Asian (13.5% versus 23.2%), Black (4.4% versus 9.4%), Arab (3.8% versus 6.2%), and Indigenous (2.3% versus 4.1%) ethnicities. There were no significant differences in percentages of Filipino, Chinese, Latin America or mixed ethnic group populations, between those with complete versus missing covariate data. In terms of oral health outcome variables, the percentage of children having at least two teeth with untreated caries (outcome 3) was significantly lower among those with complete data, compared to those with missing covariate data, (16.5% vs 20.9%). There were no other differences between those with complete versus missing covariate data on the other oral health outcome variables.

Sample size is often calculated before the study to identify the minimum sample size to reject the null hypothesis with desired power and statistical significance. However, in our study, we have tried to recruit all the grade 1 and/or 2 students in our study population and aimed to assess the effect of ethnicity on children’s oral health outcomes without a predetermined hypothesis since this study is exploratory/non-hypothesis driven in nature. Therefore, our main consideration for sample size is the stability of model, which indicates whether the model parameters can be estimated from the data.

In logistic regression (our study used the multivariable logistic regression), a common used rule to determine the stability of model estimation is the ratio of minimum (events, non-events) to number of candidate parameters in the model. Among our health outcomes, the outcome of parent-rated fair or poor oral health status has the most unbalanced distribution of outcome (10.4% of *n* = 5600 reported fair or poor oral health status, shown in Table 1). In our most complex model (i.e., fully adjusted model (model 3)), we had 20 parameters (including intercept parameter). The calculated ratio in our study is around 29 (5600*0.104/20 = 29.12), which is higher than the suggested value of at least 20, which were suggested by van der Ploeg et al. [36]. Therefore, our study had enough power to get the stable estimate from the model.

**Table 1:** Descriptive statistics (mean or %) of weighted sample: oral health outcomes, socio-demographic and caries-related behaviours variables, by ethnic group

Variables	Ethnic groups										
	All	White	South Asian	Filipino	Chinese	Black	Arab	Latin America	Indigenous	Mixed ethnic	*P*-value
	(*n* = 5600)	(*n* = 2944)	(*n* = 771)	(*n* = 345)	(*n* = 301)	(*n* = 241)	(*n* = 193)	(*n* = 166)	(*n* = 95)	(*n* = 544)	
Oral health outcomes											
Children with dental caries, %	56.7	49.2	60.2	76.1	70.0	58.3	81.9	54.1	81.9	57.0	<0.001
Average count of deft/DMFT	2.71	2.0	2.9	5.2	3.7	2.8	4.7	2.2	5.5	2.8	<0.001
Average count of deft/DMFT if deft/DMFT > 0	4.76	4.0	4.9	6.9	5.2	4.9	5.7	4.1	6.7	4.9	<0.001
≥ 2 teeth with untreated caries, %	16.5	9.6	25.0	38.3	18.1	23.9	29.5	15.9	32.4	14.9	<0.001
Parent-rated oral health (fair or poor), %	10.4	7.5	14.2	14.8	20.3	8.8	14.2	11.1	22.3	8.8	<0.001
Sociodemographic variables											
Age (≥ 7 years), %	74.7	75.3	71.8	82.6	72.0	69.5	77.7	71.8	71.2	74.4	0.025
Male, %	49.6	50.2	48.2	48.2	50.7	45.1	54.7	44.4	44.5	51.6	0.201
Having dental insurance, %	84.7	89.6	72.3	85.3	85.5	74.2	75.0	73.6	89.3	85.7	<0.001
Highest level of educational attainment in household (high school diploma or less), %	14.8	12.3	14.6	7.0	15.5	32.5	22.6	17.2	40.8	14.7	<0.001
Dwelling ownership of family (non-owned), %	30.1	21.6	35.6	43.4	9.8	64.2	51.2	54.0	78.7	27.2	<0.001
Caries-related behaviors											
Brushing teeth twice or more times per day, %	62.8	62.8	55.4	90.8	70.6	67.4	32.9	67.6	36.7	65.6	<0.001
Sugar sweetened beverage consumption (high level), %	23.0	10.3	41.1	41.1	15.6	58.8	48.1	37.9	53.2	20	<0.001
One or more routine dental visits in the past year, %	77.2	85.3	54.3	69.8	83.8	64.2	66.9	66.2	63.7	83.2	<0.001

NOTE: for the purpose of summarizing results in the text, we use the term “visible minority” to describe all groups except White and Indigenous [10]. We also considered the mixed ethnicity category separately

Across our study sample (*n* = 5600), 74.7% of children were age 7 or 8 years. The sex breakdown was similar across ethnic groups (nearly half males). Ethnicity was distributed across our sample as follows: 51.8% White, 13.4% South Asian, 6.2% Filipino, 5.1% Chinese, 4.4% Black, 3.8% Arab, 3.1% Latin America, 2.3% Indigenous and 9.9% mixed ethnic groups. For purpose of comparison with population estimates (the 2011 National Household Survey), we also examined the ethnicity distribution across the full sample (i.e., sample including those whose ethnicity fell into a smaller group (*n* < 100), and those whose ethnicity was recorded as “other” and could not be re-classified into an existing category, see Additional file 2: Figure S1). The percentages of different ethnic groups in our full sample versus the 2011 National Household Survey (NHS) for the same geographic regions (i.e., Calgary and Edmonton combined) were: White: 49.5% (our sample) versus 59.2% (NHS); South Asian: 12.8% versus 9.0%; Filipino: 5.9% versus 3.8%; Chinese: 4.9% versus 3.9%; Black: 4.2% versus 4.4%; Arab: 3.7% versus 2.3%; Latin America: 2.9% versus 1.9%; Indigenous: 2.2% versus 5.9%; and mixed ethnic group: 9.4% versus 6.1%.

### Descriptive statistics for oral health outcomes and covariates

Table 1 shows the distribution of oral health outcomes, sociodemographic variables and caries-related behaviors across different ethnic groups. Overall, 56.7% of children were affected by dental caries, and the average number of teeth with caries experience (deft/DMFT) was 2.71 (2.59 for primary (deft) and 0.12 for permanent (DMFT)). For children with dental caries experience (deft/DMFT > 0), the average number of teeth with dental caries was 4.76 (4.55 for deft and 0.21 for DMFT). 16.5% of children had at least 2 teeth with untreated caries (d or D), and 10.4% of children were rated by their parents as having “fair or poor” health of teeth and mouth.

Table 1 also shows that there were significant differences between ethnic groups in all oral health outcomes, caries-related behaviors and socioeconomic factors (Table 1). In particular, Filipino, Arab, and Indigenous children had the worst clinician-assessed oral health outcomes (i.e., dental caries prevalence, average count of deft/DMFT, and percent with 2 or more teeth with untreated caries) while Chinese children were most likely to have “fair or poor” oral health ratings from their parents. Based on parental reports, South Asian, Latin America, Black, and Arab children had lower rates of having dental insurance (72.3%, 73.6%, 74.2% and 75.0%, respectively) compared to other ethnic groups. In terms of household educational attainment, Indigenous and Black children were most likely (40.8% and 32.5%, respectively) and Filipino children were least likely (7.0%) to fall into the lowest household educational attainment category (high school diploma or less). Indigenous and Black children were most likely (78.7% and 64.2% respectively) and Chinese children were least likely (9.8%) to live in homes that were not owned by their families. Filipino children were most likely (90.8%) to brush their teeth at least twice per day, while Chinese and White children had the lowest consumption frequency of sugar-sweetened beverages (15.6% and 10.3% with reported consumption of 4 or more times per week, respectively). Just over half (54.3%) of South Asian children had one or more routine dental visits in the past year, which was much lower than the other ethnic groups.

### Regression analysis

Table 2 shows the results of binary logistic regression and zero-inflated negative binomial (ZINB) regression analyses. Ethnic disparities were apparent for all four oral health outcomes. The predominant pattern was that most visible minority populations, except for Latin American children, had worse oral health outcomes than White populations. Indigenous and mixed ethnic groups also had worse oral health outcomes than White populations. Overall, most effects attenuated but persisted (remained statistically significant) with consecutive adjustment for blocks of covariates (i.e., from model 1 to 3). Focusing on the fully-adjusted models (model 3), for outcome 1 (dental caries prevalence), children in South Asian, Filipino, Chinese, Arab, Indigenous and mixed ethnic groups were significantly more likely to have caries experience (deft/DMFT > 0) compared to White children, whereas Black and Latin American children did not significantly differ from White children. For outcomes 2 and 3 (i.e., average count of deft/DMFT and 2 or more teeth with untreated caries, respectively), most visible minority groups (except for Latin American children), as well as Indigenous children and children classified as mixed ethnicity, were significantly more likely to have poorer oral health outcomes than White children after full adjustment. For outcome 4 (parent-rated fair or poor oral health status), South Asian, Filipino, Chinese and Indigenous children were more likely to have parent ratings of “fair or poor” oral health than White children, whereas Black, Arab, Latin American and mixed ethnic children did not differ significantly from White children.

**Table 2:** Associations (odds ratio (OR) or incidence rate ratio (IRR)) between oral health outcomes and ethnicity identity, adjusting for increasing number of covariates

Models	Ethnic groups (reference group: White)							
	South Asian	Filipino	Chinese	Black	Arab	Latin America	Indigenous	Mix ethnic
Outcome 1: Children with dental caries - OR (95% CI)								
Model 1	1.55 (1.32, 1.82)	3.52 (2.82, 4.39)	2.37 (1.92, 2.93)	1.50 (1.17, 1.92)	3.80 (2.82, 5.14)	1.16 (0.90, 1.51)	4.52 (2.96, 6.90)	1.33 (1.14, 1.56)
Model 2	1.50 (1.28, 1.76)	3.40 (2.70, 4.28)	2.51 (2.02, 3.12)	1.26 (0.95, 1.67)	3.38 (2.51, 4.55)	1.12 (0.87, 1.45)	3.96 (2.50, 6.28)	1.32 (1.12, 1.56)
Model 3	1.51 (1.27, 1.79)	3.18 (2.48, 4.08)	2.61 (2.07, 3.28)	1.15 (0.83, 1.59)	3.71 (2.67, 5.17)	1.11 (0.84, 1.46)	3.43 (2.11, 5.59)	1.33 (1.12, 1.58)
Outcome 2: Caries experience (count of deft/DMFT) - IRR (95% CI)								
Model 1	1.21 (1.13, 1.30)	1.83 (1.71, 1.95)	1.34 (1.24, 1.44)	1.24 (1.12, 1.37)	1.50 (1.35, 1.66)	1.05 (0.91, 1.21)	1.83 (1.64, 2.05)	1.25 (1.17, 1.35)
Model 2	1.21 (1.13, 1.30)	1.80 (1.69, 1.93)	1.38 (1.28, 1.49)	1.15 (1.03, 1.27)	1.41 (1.27, 1.56)	1.00 (0.87, 1.16)	1.61 (1.43, 1.82)	1.24 (1.15, 1.33)
Model 3	1.23 (1.14, 1.32)	1.80 (1.66, 1.94)	1.40 (1.30, 1.51)	1.13 (1.01, 1.28)	1.36 (1.22, 1.52)	0.99 (0.85, 1.15)	1.58 (1.39, 1.79)	1.23 (1.14, 1.32)
Outcome 3: ≥ 2 teeth with untreated caries – OR (95%CI)								
Model 1	3.04 (2.53, 3.65)	6.14 (4.95, 7.62)	2.14 (1.65, 2.76)	3.00 (2.29, 3.94)	3.66 (2.57, 5.22)	1.68 (1.18, 2.39)	3.96 (2.48, 6.34)	1.60 (1.25, 2.05)
Model 2	2.63 (2.17, 3.17)	5.50 (4.42, 6.86)	2.34 (1.82, 3.03)	2.21 (1.64, 2.98)	2.92 (2.07, 4.13)	1.33 (0.93, 1.91)	2.51 (1.45, 4.36)	1.50 (1.16, 1.93)
Model 3	1.95 (1.58, 2.40)	4.77 (3.71, 6.13)	2.41 (1.84, 3.15)	1.62 (1.15, 2.28)	2.17 (1.47, 3.20)	1.13 (0.78, 1.65)	2.00 (1.11, 3.61)	1.49 (1.15, 1.92)
Outcome 4: Fair or poor oral health status – OR (95%CI)								
Model 1	2.08 (1.70, 2.55)	2.13 (1.64, 2.77)	3.24 (2.52, 4.16)	1.32 (0.93, 1.87)	2.03 (1.38, 3.00)	1.50 (1.06, 2.13)	4.58 (3.11, 6.74)	1.25 (0.98, 1.60)
Model 2	1.83 (1.49, 2.25)	2.00 (1.51, 2.66)	3.65 (2.80, 4.75)	0.91 (0.63, 1.33)	1.44 (0.96, 2.16)	1.16 (0.82, 1.65)	3.24 (2.18, 4.82)	1.19 (0.92, 1.54)
Model 3	1.52 (1.20, 1.94)	2.25 (1.64, 3.09)	3.97 (3.02, 5.20)	0.86 (0.56, 1.31)	1.05 (0.65, 1.68)	1.20 (0.83, 1.72)	2.43 (1.57, 3.77)	1.25 (0.96, 1.63)
Covariates adjustment:								
Model 1: age, sex and city.								
Model 2: Model 1 covariates + highest level of educational attainment, dwelling ownership, and dental insurance.								
Model 3: Model 2 covariates + frequency of tooth-brushing/day, routine dental visit in past year, sugar sweetened beverage consumption.								

NOTE: for the purpose of summarizing results in the text, we use the term “visible minority” to describe all groups except White and Indigenous [10]. We also consider the mixed ethnicity category separately

For some ethnic groups, the parent-rated outcome (i.e., outcome 4) appeared somewhat inconsistent with the clinician-assessed outcomes (i.e., outcomes 1-3). Specifically, although we did not directly compare across ethnic groups, we noted that Filipino children had the highest coefficients for untreated dental problems (e.g., OR = 4.77 relative to Whites, see Table 2, outcome 3, model 3), while their coefficients for parent-rated oral health were more similar to those of other ethnic groups (e.g., OR = 2.25 relative to Whites, see Table 2, outcome 4, model 3). On the other hand, Chinese children had the highest coefficients for parent-rated oral health (e.g., OR = 3.97 relative to Whites, see Table 2, outcome 4, model 3), while their coefficients for untreated dental problems were more similar to those of other ethnic groups (e.g., OR = 1.84 relative to Whites, see Table 2, outcome 3, model 3).

Focusing on model 3 (fully adjusted by all covariates), the c statistics for outcomes 1, 3, and 4, using logistic regression, are 0.63, 0.73, and 0.67 respectively, which indicates modest discrimination ability. The root mean square error (RMSE) for zero-inflated negative binomial (ZINB) model on the outcome 2 is 3.18.

## Discussion

The results of this study show that ethnic disparities in children’s oral health were present in the urban settings of Calgary and Edmonton, Canada. Among grade 1 and 2 schoolchildren, members of certain visible minority groups (i.e., South Asian, Filipino, Chinese, Black, Arab), as well as members of Indigenous, and mixed, ethnic groups, were more likely to have dental caries experience (deft/DMFT) and untreated dental problems, compared to children identified as White. Members of Filipino, Arab and Indigenous groups were particularly affected. One exception was Latin American children, who did not differ significantly from White children on oral health outcomes. Visible minority and Indigenous populations in our sample tended to have lower socioeconomic status and poorer caries-related behaviors compared to White populations. However, even after adjusting for these covariates, most of the significant ethnic disparities in oral health persisted. Perhaps most notably, in fully adjusted models (model 3), Filipino children remained far more likely than White children to have untreated dental problems (outcome 3). The reasons for the particularly large effects observed in the Filipino children in our study are unknown but their magnitude merits further study.

Socioeconomic factors are strong determinants of oral health as documented in both Canadian and international literature [7, 37, 38]. It is also well established that visible minority groups and Indigenous people are more likely to experience lower socioeconomic position compared to White populations [39]. Therefore, it is important to understand whether or the extent to which the socioeconomic factors could account for observed ethnic disparities in children’s oral health. Overall, a large body of U.S. research shows that socioeconomic factors could account for much of the oral health differences between Black and White populations, and in fact they fully accounted for Black / White differences in some studies [18, 21]. In some contrast, in our study, the socioeconomic factors accounted for only a small part of the ethnic disparities in oral health (i.e., results from model 1 to 2). The differences between the two countries may reflect, in part, that axes of ethnic identity and socioeconomic status are more independent of one another in Canada than in the U.S. They could also reflect methodological limitations of our study, which are discussed in more detail below.

Evidence exists to suggest that certain behaviors are important risk factors for developing dental caries [40]. The results of our study show that there were significant ethnic disparities in some oral health-related behaviors, such as daily tooth-brushing and sugar-sweetened drink consumption. After adjusting for those behaviors, ethnic disparities in oral health outcomes were attenuated but did not disappear. These findings suggest that, in Canada, there must be other potential factors driving ethnic disparities in oral health, which were not included in our study. Examples might include aspects of a cariogenic diet other than sugar-sweetened beverages, such as consumption of candy or night time meals or drinks, which have been shown to increase the risk of developing dental caries [40], or practice and duration of infant feeding (i.e., breast, bottle or mixed). For example, studies have shown that breast-fed children were less affected by dental caries than bottle-fed children [31] and children who were never breast-fed or who were breast-fed for more than 24 months had a higher prevalence of dental caries, compared to children who were breast-fed for 24 months or less [17, 40, 41].

This study included both clinician-assessed and parent-rated outcomes of oral health. Clinical data are important for disease identification, while parent perception measures may reflect or influence oral health-related behaviors (including visits to dental professionals) and in turn, oral health itself [42]. Our results showed, in some cases, apparent inconsistencies between the two types of measures, specifically within Filipino and Chinese children. Understanding the differences in meaning and implications of clinician-assessed and parent perception measures may shed light on steps that need to be taken to improve oral health in the different ethnic groups.

Even though the number of Indigenous children in this study was smaller than other ethnic groups and fell below our working cut-off (*n* < 100), we included them as a distinct group because of their unique cultural heritage and identity in Canada [32]. Other research has shown that Indigenous people experience higher rates of dental caries than other Canadians [33], and these inequalities are evident for both Indigenous persons living in urban areas and those living in remote First Nations and Inuit communities [7, 43, 44]. This study only sampled Indigenous children who were living in urban areas (Calgary and Edmonton) and results showed that among those children, 81.9% had dental caries experience (deft/DMFT > 0) and an average of 5.5 teeth (either primary or permanent) were affected by dental caries. These estimates are slightly lower than national estimates from the Canadian Health Measures Survey (CHMS, 2007-09), where 89.2% of Indigenous children aged 6-11 years (excluding those who was living on reserves) had caries experience, and there was an average of 6.62 teeth (either primary or permanent) affected by dental caries [7]. The difference in age group between our sample and that of the CHMS could partly explain the differences. Additional research on reasons and potential solutions for poor oral health among Indigenous Canadians is a very important priority.

This research has limitations. First, these data are cross-sectional. Although we can rule out ethnicity being caused by oral health indicators, conclusions about causal relationship cannot be drawn in terms of potentially important covariates unmeasured in this study, such as infant feeding practices.

Second, amongst the five assessment teams who collected the mouth examination data for this study, inter- and intra-examiner reliability is unknown. Although all teams undertook consistent calibration, within a short window of time, and by the same expert calibrator; inconsistencies within and between raters could influence the validity and reliability of our findings. Third, because our study is a secondary analysis of data collected for another purpose, we were unable to include other variables that may have been uniquely important for the present study; examples may include infant feeding practices, toddlers’ age at first tooth, or night time meals or drinks. These factors have been found to have a significant association with dental caries at school age in other research [45] and may help explain the remaining ethnic disparities observed here.

Fourth, some of our variables had limitations, for example, our dental insurance indicator combined public and private forms of insurance, which can differ substantially in terms of coverage [8]. The indicator of one or more routine dental visits in the past year conflates preventive and treatment activities that may have taken place during the visit, and there may be important differences between the two. Ethnic identity, like all self-reported items, may be subject to some reporting bias. In our study, however, ethnicity is self-identified and we included ‘select all that apply’ as well as an open-ended ‘other’ category, which theoretically should improve respondents’ ability to convey the ethnic group(s) with which they align. Other reported variables, such as dental hygiene behaviours, may be subject to social desirability bias; however, the anonymous nature of our survey would be expected to reduce that bias to some extent. Fifth, minority groups of Southeast Asian, Korean and Japanese children were not included in our study due to small numbers in our sample. We made the decision to group together those children whose parents selected more than one single ethnic group (i.e., mixed ethnic group); we recognize that that group would have had significant, though not easily interpretable, heterogeneity. Lastly, based on response rates, one might be concerned about bias in our sample. However, examination of participation rates by income quartile, school system, and geographic area did not reveal any obviously biased patterns.^2^ Also, post stratification weights by income quartile were applied to data analysis to address participation disparities by participants’ socioeconomic status.

A strength of the study is its novelty: we are not aware of any other published Canadian reports on ethnic disparities in oral health in a population-based sample of children that include a mouth examination. In light of its inclusion of both clinician-assessed oral health data and ethno-cultural information available, this study is the first to report the ethnic disparities in oral health in Canada.

## Conclusions

This population-based, cross-sectional study demonstrated that ethnic disparities in children’s oral health exist in Canada; specifically, among grade 1 and 2 schoolchildren in urban settings in the province of Alberta. Adjustment for demographic, socioeconomic, and caries-related behavior variables attenuated but did not eliminate the observed ethnic disparities in oral health. These findings suggest that there are additional, more complex factors driving ethnic disparities in oral health and further research is needed to explore potential remaining contributors to such disparities, which may be a combination of cultural, biological, environmental, behavioral, and lifestyle-related factors. Further research may benefit from other analytic methods such as path analysis, to sort out potential mediating and moderating relationships amongst axes of stratification (e.g., ethnicity, socioeconomic circumstances), covariates (e.g., age, sex, demographic and behavioral variables), and oral health outcomes. Our findings provide an opportunity to inform universal and targeted preventive and treatment-based public oral health programs.

## Additional files

Additional file 1: Appendix A. Question about participants’ ethnic identity in parent questionnaire: Shows the exact question that was asked in the parent questionnaire about the child’s ethnic identity (DOCX 12 kb)

Additional file 2: Figure S1. Flow chart of participant exclusions. Shows the flow chart of participant exclusions, from the initial sample with data available from both the open mouth exam and the parent questionnaire, to the final analytic sample which takes into account missing data and exclusions for other reasons (e.g., ethnic identity grouping that fell below our cut-off of *n*=100). * Main sources of missing covariate data were: sugar-sweetened beverage consumption (*n*=317), at least one routine dental visit in the past year (*n*=184), dwelling ownership (*n*=146), household educational attainment (*n*=127), and sex (*n*=134); other covariates in this study had 10 or fewer missing cases. These numbers total more than 792 due to children with missing data on multiple covariates. (DOCX 27 kb)

## Abbreviations

95% CI: 95% confidence interval
deft/DMFT: The number of decayed (d/D), extracted/missing (e/M, due to decay) and filled (f/F) teeth (deft-primary teeth, DMFT-permanent teeth)
OR: Odds ratio

## Footnotes

1 Although other previously published Canadian studies, including the Canadian Health Measures Survey (CHMS), used the term “Aboriginal”, we elected to use the term “Indigenous” because the Government of Canada is now using that term to identify peoples of First Nations, Inuit and Métis heritage (https://www.aadnc-aandc.gc.ca/).

2 For example, participation rates by school system were 54.7% (Public) and 61.7% (Catholic) in Calgary; and 57.5% (Public) and 46.2% (Catholic) in Edmonton. School-level participation rates by income quartile were 69.1% (Q1), 67.3% (Q2), 49.1% (Q3), and 43.6% (Q4) in Calgary; and 53.7% (Q1), 47.3% (Q2), 63.6% (Q3), and 51.9% (Q4) in Edmonton. Student-level participation rates by income quartile, within participating schools, were: 44.6% (Q1), 47.7% (Q2), 50.3% (Q3), and 54.0% (Q4) in Calgary; and 36.0% (Q1), 43.2% (Q2), 51.0% (Q3), and 51.8% (Q4) in Edmonton.
