# Supplementary material for: Ethnic disparities in children’s oral health: findings from a population-based survey of grade 1 and 2 schoolchildren in Alberta, Canada
Source: BMC Oral Health. 2018 Jan 4;18:1. doi: 10.1186/s12903-017-0444-8 (PMC5753483; doi:10.1186/s12903-017-0444-8)
Supplement: Supplementary file 1 — Appendix A. Question about participants’ ethnic identity in parent questionnaire: Shows the exact question that was asked in the parent questionnaire about the child’s ethnic identity (DOCX 12 kb) [file 12903_2017_444_MOESM1_ESM.docx]

**APPENDIX A**

Question about participants’ ethnic identity in parent questionnaire:

Please tell us about your ethno-cultural background (please select all that apply):

- First Nation
- Metis
- Inuit
- White
- Chinese
- South Asian (e.g., India, Bangladesh, Pakistan, Sri Lanka)
- Black
- Filipino
- Latin America
- Southeast Asian (e.g., Cambodia, Indonesia, Laos, Vietnam)
- Arab
- West Asian (e.g., Afghanistan, Iran)
- Japanese
- Korean
- Other, please specify________________________________
