# Supplementary material for: Ethnic disparities in children’s oral health: findings from a population-based survey of grade 1 and 2 schoolchildren in Alberta, Canada
Source: BMC Oral Health. 2018 Jan 4;18:1. doi: 10.1186/s12903-017-0444-8 (PMC5753483; doi:10.1186/s12903-017-0444-8)
Supplement: Supplementary file 2 — Flow chart of participant exclusions. Shows the flow chart of participant exclusions, from the initial sample with data available from both the open mouth exam and the parent questionnaire, to the final analytic sample which takes into account missing data and exclusions for other reasons (e.g., ethnic identity grouping that fell below our cut-off of n=100). * Main sources of missing covariate data were: sugar-sweetened beverage consumption (n=317), at least one routine dental visit in the past year (n=184), dwelling ownership (n=146), household educational attainment (n=127), and sex (n=134); other covariates in this study had 10 or fewer missing cases. These numbers total more than 792 due to children with missing data on multiple covariates. (DOCX 27 kb) [file 12903_2017_444_MOESM2_ESM.docx]

n=6,884 with data from both open mouth exam and parent questionnaire

n=45 excluded for missing data on outcome variable (parent-rated oral health in all cases)

n=131 excluded for missing data on ethnic identity

n=15 excluded for ethnic group recorded as ‘other’, which could not be reclassified into an existing category

n=301 excluded because the ethnic group selected fell below our cutoff (<100) of group size needed for analysis

n=792 excluded for missing data on other covariates*

n=5,600 analytic sample
